# DNMT1 mediates chemosensitivity by reducing methylation of miRNA-20a promoter in glioma cells

**DOI:** 10.1038/emm.2015.57

**Published:** 2015-09-04

**Authors:** Daoyang Zhou, Yingfeng Wan, Dajiang Xie, Yirong Wang, Junhua Wei, Qingfeng Yan, Peng Lu, Lianjie Mo, Jixi Xie, Shuxu Yang, Xuchen Qi

**Affiliations:** 1 Department of Emergency, Sir Run Run Shaw Hospital, College of Medical Sciences, Zhejiang University, Hangzhou, China; 2 Department of Neurosurgery, Sir Run Run Shaw Hospital, College of Medical Sciences, Zhejiang University, Hangzhou, China; 3 Department of Intensive Care Unit, Sir Run Run Shaw Hospital, College of Medical Sciences, Zhejiang University, Hangzhou, China; 4 College of Life Science, Zhejiang University, Hangzhou, China

## Abstract

Although methyltransferase has been recognized as a major element that governs the epigenetic regulation of the genome during temozolomide (TMZ) chemotherapy in glioblastoma multiforme (GBM) patients, its regulatory effect on glioblastoma chemoresistance has not been well defined. This study investigated whether DNA methyltransferase (DNMT) expression was associated with TMZ sensitivity in glioma cells and elucidated the underlying mechanism. DNMT expression was analyzed by western blotting. miR-20a promoter methylation was evaluated by methylation-specific PCR. Cell viability and apoptosis were assessed using the 3-(4,5-dimethyl-2-thiazolyl)-2,5-diphenyl-2-H-tetrazolium bromide (MTT) and TdT-mediated dUTP-biotin nick end labeling assays, respectively. The results showed that compared with parental U251 cells, DNMT1 expression was downregulated, miR-20a promoter methylation was attenuated and miR-20a levels were elevated in TMZ-resistant U251 cells. Methyltransferase inhibition by 5-aza-2′-deoxycytidine treatment reduced TMZ sensitivity in U251 cells. In U251/TM cells, DNMT1 expression was negatively correlated with miR-20a expression and positively correlated with TMZ sensitivity and leucine-rich repeats and immunoglobulin-like domains 1 expression; these effects were reversed by changes in miR-20a expression. DNMT1 overexpression induced an increase in U251/TM cell apoptosis that was inhibited by the miR-20a mimic, whereas DNMT1 silencing attenuated U251/TM cell apoptosis in a manner that was abrogated by miR-20a inhibitor treatment. Tumor growth of the U251/TM xenograft was inhibited by pcDNA-DNMT1 pretreatment and boosted by DNMT1-small hairpin RNA pretreatment. In summary, DNMT1 mediated chemosensitivity by reducing methylation of the microRNA-20a promoter in glioma cells.

## Introduction

Glioblastoma multiforme (GBM) is the most common malignant primary brain tumor in adults and is one of the most aggressive human tumors. Its therapeutic schemes represent a difficult problem for patients. At present, alkylating agents are the most popular and effective drugs for GBM chemotherapy. A bioavailable imidazotetrazine derivative of dacarbazine (temozolomide; TMZ) easily penetrates the blood–brain barrier^1^ and has been demonstrated to possess broad-spectrum antitumor activity chemotherapeutic following oral administration. TMZ can efficiently inhibit the proliferation of glioma cells and induce apoptosis.^2^ In systemic circulation, TMZ undergoes rapid chemical decomposition to its active compound MTIC(5-(3-methyltriazen-1-yl) imidazole-4-carboximide) and subsequently causes guanine methylation at the O^6^-position.^3^ In turn, this modification yields DNA-alkylating species and leads to cytotoxicity. However, the development of chemoresistance can result in an unsatisfactory outcome of TMZ-chemotherapy.^1^

DNA methylation mediated by DNA methyltransferases (DNMTs) is one of major mechanisms that govern the epigenetic regulation of the genome. Aberrant epigenetic inactivation of tumor suppressor genes leads to gene silencing, followed by subsequent deregulation of various signaling pathways in a number of human malignancies. This gene silencing is characterized by DNA hypermethylation of promoter regions.^4^ In mammals, DNA methylation is maintained by DNMTs such as DNMT1, DNMT3a and DNMT3b via the transfer of a methyl group to the 5-carbon in the cytosine of a CpG dinucleotide. Accumulating evidence has demonstrated that DNMTs mediate transcriptional silencing in malignant gliomas.^5^ DNMTs associated with chemoresistance have also been found in various cancers. Inhibition of DNMT1 has been proposed as an adjuvant therapeutic approach to overcome ovarian cancer chemoresistance.^6^ However, the effect of DNMT regulation on glioblastoma chemoresistance has not been well defined.

microRNAs (miRNAs) are endogenous 22-nucleotide non-coding RNAs that regulate the expression of target genes by degrading target messenger RNA (mRNA) transcripts and inhibiting mRNA translation.^7^ Since their discovery, >1000 human miRNAs have been identified.^8^ In previous decades, a large number of studies demonstrated that the biogenesis and regulatory machinery of miRNAs had important roles in the development and progression of various types of cancer, including malignant glioma.^9^ The primary focus of these studies has been on the role of miRNAs in drug resistance in cancer. For example, miR-20 was demonstrated to be involved in leukemia^10^ and colorectal adenocarcinoma.^11^ Therefore, we investigated whether there was a link between miR-20a expression and glioma chemoresistance. Knowledge of how miRNAs are regulated in complex gene regulatory systems has attracted considerable attention. The genetic regulation of miRNAs is similar to the regulation of mRNAs and involves specific transcription factors or proteins that interact with the promoter.^12, 13^ A computer-assisted approach indicated that 46 potential miRNAs located in the human imprinted 14q32 domain were embedded within a conserved CpG island locus that could be regulated by DNA methylation.^14^ Another example is mir-34b/c and mir-124a, which have been shown to have a CpG island near their promoter region.^15, 16^

Because CpG methylation of the proximal promoter is the epigenetic mechanism regulating gene expression, we designed this study to determine whether miR-20a promoter CpG methylation was related to DNMT1 expression. We investigated whether CpG methylation was involved in glioblastoma resistance to TMZ and attempted to elucidate the underlying mechanism.

## Materials and Methods

### Reagents

TMZ was provided by the Tasly Pharmaceutical Co., Ltd (Tianjin, China) and freshly dissolved in 10% dimethylsulfoxide (Sigma-Aldrich, St Louis, MO, USA). The dimethylsulfoxide concentration was kept below 0.1% in all cell culture experiments because at this concentration dimethylsulfoxide did not exhibit any detectable effect on cell growth or viability. The 5-aza-2′-deoxycytidine (5-aza-dC) methyltransferase inhibitor was purchased from Sigma-Aldrich. Anti- leucine-rich repeats and immunoglobulin-like domains 1 (LRIG1) antibody was purchased from Santa Cruz Biotechnology, Inc. (Santa Cruz, CA, USA), and the anti-DNMT1, anti-DNMT3a and anti-DNMT3b antibodies were obtained from IMGENEX (San Diego, CA, USA).

### Cell culture

The human glioblastoma cell line U251 was purchased from Sigma and routinely cultured in Dulbecco's modified Eagle media (GIBCO, Life Technologies, Helgerman Court, MD, USA) under condition of 5% CO2 at 37 °C. To establish the resistant cell line, U251 cells were exposed to stepwise increasing concentrations of TMZ (1.25–80 μmol) over a period of 8 months. After incubation, the selected cell were approximately sevenfold more resistant to TMZ compared with the parental U251 cells. The resistant cell line is referred to as U251/TM throughout the article. The cells were challenged frequently with 80 μmol TMZ to maintain their resistant phenotype.

### Methylation-specific PCR and quantitative real-time PCR

RNA was extracted from glioblastoma cells using the TRIzol reagent for LRIG1 miRNA analysis or the TRIzol SM reagent for miR-20a analysis (Invitrogen, Carlsbad, CA, USA) according to the manufacturer's protocols. The methylation status of the miR-20a promoter was quantitated using the methylation-specific PCR method. First, genomic DNA from the cultured cells and specimens was isolated using a Universal Genomic DNA Extraction Kit Ver3.0 (Takara Bio Inc., Tokyo, Japan). The DNA concentration and purity were assessed by ultraviolet-visible spectrophotometry (1.8, A260/A280, 2.0). Then, the genomic DNA of each sample was treated with bisulfite modification as follows: genomic DNA was denatured in 3 mol l^-1^ NaOH for 15 min at 37 °C, and the cytosines were sulfonated in 3.6 mol l^-1^ sodium bisulfite and 1 mmol l^-1^ hydroquinone (Sigma-Aldrich) for 16 h at 55 °C. The modified DNA samples were desalted using a DNA clean-up system (Promega Corporation, Fitchburg, WI, USA). The bisulfite-treated genomic DNA was amplified by Takara's Ex Taq DNA Polymerase using two primers for miR-20a-1 and miR-20a-2 that covered almost the entire CpG-rich region of the proximal miR-20a promoter. The bisulfite PCR products were sequenced for 9–12 reactions by the Biomedical Instrumentation Center (USUHS, USA). Methylation-specific PCR products were analyzed in a 2% agarose gel stained with ethidium bromide. The miR-20a promoter was normalized to non-methylated 16 S RNA.

The expression of miR-20a and LRIG1 mRNA were determined using quantitative real-time PCR. miR-20a expression was quantified using the NCodeTM miRNA First-Strand cDNA Synthesis Kit MiR-20a (Invitrogen) and the KAPATM SYBR FAST qPCR Kit (Kapa Biosystems, Wilmington, MA, USA), with miR-16 as an internal control to normalize the complementary DNA input according to the manufacturer's protocols.^17^ miR-20a expression was normalized to the small nuclear RNA RNU6B. The expression of the LRIG1 mRNA was determined using the LightCycler 480 Probes Master Kit (Roche Diagnostics, Indianapolis, IN, USA) according to the manufacturer's instructions. All quantitative real-time PCRs were run in a LightCycler 480 System II (Roche Diagnostics) and were performed in triplicate.

### Western blot assay

Protein expression was analyzed by western blotting. First, after washing with ice-cold phosphate-buffered saline, the cells were lysed in radioimmunoprecipitation assay lysis buffer (25 mmol l^-1^ 4-(2-hydroxyethyl)-1-piperazineethanesulfonic acid, pH 7.5, 50 mmol l^-1^ NaCl, 1% NP40, 2.5 mmol l^-1^ ethylenediaminetetraacetic acid, 10% glycerol and 1% triton X-100) containing protease inhibitors (2 mmol l^-1^ NaF, 2 mmol l^-1^ sodium orthovanadate, 2 mmol l^-1^ sodium pyrophosphate and 1 mmol l^-1^ protein inhibitor) and incubated at 4 °C for 30 min. The cell lysates were centrifuged at 14 000 *g* for 10 min at 4 °C. The total protein was harvested from the supernatant, and the protein concentrations were determined using a BCA protein assay kit. The proteins were separated on 10% sodium dodecyl sulfate–polyacrylamide gels, then transferred to PVDF membranes (Millipore, Billerica, MA, USA) using the Bio-Rad gel system. The membranes were blocked at room temperature for 2 h with 5% nonfat milk in TBST (25 mM Tris–HCl, 50 mM NaCl, and 0.05% Tween 20), and then probed with primary antibodies (anti-DNMT1, -DNMT2, -DNMT3 and -LRIG1, 1:200 dilution; anti-β-actin, 1:1000 dilution) overnight at 4 °C. Following extensive washing, the membranes were incubated for 2 h with a fluorescent secondary antibody at a 1:5000 dilution in blocking solution (Alexa Fluor 680-conjugated goat anti-rabbit antibody, Molecular Probes, Rockland Immunochemicals, PA, USA). The probed proteins were visualized using an ECL kit according to manufacturer's protocol, and the images were analyzed by the software program from the motored molecular imaging system (ChampGel 6000; Beijing Sage).

### miRNA, small interfering RNA and gene expression constructs

The miR-20a mimics, miR-20a inhibitors and the miRNA mimic and miRNA inhibitor negative controls were purchased from Dharmacon (Lafayette, CO, USA). The DNMT1 small interfering RNA (DNMT-small hairpin RNA (shRNA)) and the small interfering RNA negative control were purchased from Santa Cruz Biotechnology. BLOCK-iT fluorescent oligos were purchased from Invitrogen. Aberrant DNMT1 expression in cells was investigated via pcDNA-DNMT1 transfection as previously described.^18^ Pri-DNMT1 was directly cloned from mouse genomic DNA into the pcDNA3.1 (Invitrogen, Shanghai, China) expression vector at the *Hin*dIII and *Xho*I sites using the appropriate primers. The resulting construct was named pcDNA3.1-DNMT1. The empty pcDNA3.1 vector was used as a control. DNMT1-shRNAs were purchased from Gene Pharma Co., Beijing, China. (5′-CCAUGAGCACCGUUCUCCTT-3′ and 5′-GGAGAACGGUGCUCAUGGTT-3′).

### Transfections

For plasmid transfection, U251/TM cells were transfected with the pcDNA3.1-DNMT1 plasmid or empty pcDNA3.1 using the FuGENE 6 transfection reagent (Roche Diagnostics). In brief, cells were seeded into 60 mm dishes at a density of 10^5^ cells per dish overnight prior to the transfection. Then, the transfection was performed using the FuGENE 6 Transfection Reagent (6 ml) in a 3:1 ratio with plasmid DNA (2 mg) per dish. The cells were incubated for 72 h under conditions of 5% CO2 at 37 °C.

The miR-20a inhibitor, mimic and their negative controls were transfected using lipofectamine 2000 (Invitrogen) following the manufacturer's instructions. in brief, the cells were seeded into 60 mm dishes at a density of 10^6^ cells per dish overnight prior to the transfection. The products were diluted with 50 μl Opti-MEM, and lipofectamine 2000 was diluted with an equal volume of medium. These two dilutions were mixed and then added to the cell culture plate, followed by incubation under conditions of 5% CO2 at 37 °C for 72 h. The mixed media were removed, and fresh medium was added. After transfection, the nuclear extract, whole-cell lysate, total RNA and genomic DNA were separately isolated for analysis by western blotting, quantitative real-time PCR, real-time PCR and methylation-specific PCR.

### MTT assay

U251 or U251/TM cells were pretreated with pcDNA-DNMT1, DNMT1-shRNA and/or the miR-20a mimic or miR-20a inhibitor. After 48 h of incubation, cell viability was analyzed using the MTT assay kit (Cayman Chemical Co., Ann Arbor, MI, USA) following the manufacturer's instruction. Absorption intensity was measured using an enzyme-linked immunosorbent assay reader (TECAN, Australia) at 490 nm.

### TUNEL staining assay

TMZ-induced cell apoptosis was analyzed using TdT-mediated dUTP-biotin nick end labeling staining based on the detection of labeled DNA strand breaks using an *in situ* cell detection kit (Roche Applied Science, Indianapolis, IN, USA), according to the manufacturer's instructions. In brief, the cells were fixed with formaldehyde and then permeabilized with ethanol (two changes of 100% ethanol for 3 min each, followed by 95% ethanol for 1 min) to allow penetration of the TdT-mediated dUTP-biotin nick end labeling reaction reagents into the cell nuclei. Following fixation and washing in distilled water, the incorporation of biotinylated dUTP onto the 3′ends of the fragmented DNA was accomplished in a reaction containing a terminal deoxynucleotidyl transferase. The reaction was stopped with washing buffer, and the sample was resuspended in phosphate-buffered saline containing FITC-Avidin D for 30 min at room temperature. The reaction mixture was counterstained with propidium iodide or 4',6-diamidino-2-phenylindole for 20 min. TdT-mediated dUTP-biotin nick end labeling -positive nuclei (stained brown) were counted by Motic Images Advanced 3.2 in 10 random fields ( × 200) and then averaged.

### Cell cycle detection

U251/TM cells were cultured in a 96-well plate at a density of 10^5^ cells per well to the exponential phase, then transfected with the respective oligonucleotides followed by treatment with 5 μmol l^-1^ TMZ for 24 h. Prepared 10 × BrdU solution (10 μl) was added to the plate wells in a final 1 × concentration. The mixture was incubated under conditions of 5% CO2 at 37 °C for 12 h. Following the reaction, the supernatants were removed and replaced with fresh medium. Plates containing suspension cells were centrifuged at 300 *g* for 10 min prior to the removal of the medium. Fixing/Denaturing Solution (100 μl per well) was added, and the plates were incubated at room temperature for 30 min. After fixation, the solution was removed and 1 × detection antibody solution was added (100 μl per well), followed by incubation at room temperature for 1 h. Then, prepared 1 × horseradish peroxidase-conjugated secondary antibody solution (100 μl per well) was added to the plate and incubated at room temperature for 30 min. Finally, the plates were washed three times with 1 × Wash Buffer and 100 μl of tetramethylbenzidine substrate was added, followed by an incubation for 30 min at room temperature. The reaction was shopped with 100 μl STOP solution. The absorption intensity was measured at 450 nm. For optimal readings, the plate was read within 30 min of adding the STOP Solution.

### Xenograft

In total, 4–6-week-old female C57BL/6 nu/nu mice (Beijing Animal Center) were maintained under pathogen-free conditions and provided sterile food, water and cages. The mice were maintained in the animal facility at least 1 week before the experiment. The experiments were performed in accordance with the institutional guidelines. Ambient light was controlled to provide regular cycles of 12 h of light and 12 h of darkness. For assessment of the influence of DNMT1 on glioma tumor growth, U251 cells were pre-transfected with pcDNA-DNMT1 or DNMT1-shRNA and their control plasmid. After 48 h of incubation, a suspension of 1 × 10^6^ cells in a 100 μl volume was injected subcutaneously into the right flank of the mice using a 1-cc syringe with a 27½-gauge needle. All animal procedures were performed in compliance with the Guidelines for the Care and Use of Experimental Animals established by the Committee for Animal Experimentation of Zhejiang University.

### Tumor volume measurement

When for the tumors on the flanks of the mice reached 0.5 cm, they were injected with 50 mg kg^-1^ per day TMZ for 5 days. The tumors were measured every 2 or 3 days in three perpendicular dimensions using a Vernier caliper. Tumor volumes were calculated using the modified ellipse volume formula (Volume=(height × width × depth)/2). Growth delay was calculated as the number of days required to reach a tumor volume of 1.75 cm^3^ for treatment groups relative to the control.

### Luciferase reporter assay

Cells were plated at a density of 1 × 10^4^ cells per well in 24-well plates for 24 h. After incubation, 100 ng of the pGL3-LRIG1 plasmid was co-transfected with the miR-20a mimic, inhibitor or negative control into cells using Lipofectamine 2000 (Invitrogen) for the LRIG1 3′-UTR reporter assay. The cells were incubated for 48 h and harvested for luciferase assays using the Luciferase Reporter Assay system (Promega Corporation) according to the manufacturer's protocol.

### Statistical analysis

Data were expressed as the mean±s.d. Statistical Packages for Social Sciences (SPSS 11.5) was used for data analysis. For studies involving more than two groups, data were evaluated with one-way analysis of variance followed by *post hoc* analysis with the Student–Newman–Keuls test. The level of statistical significance was set at *P*<0.05 for all cases.

## Results

### Methylation of the miR-20a promoter in glioma cells

**Figure 1:**
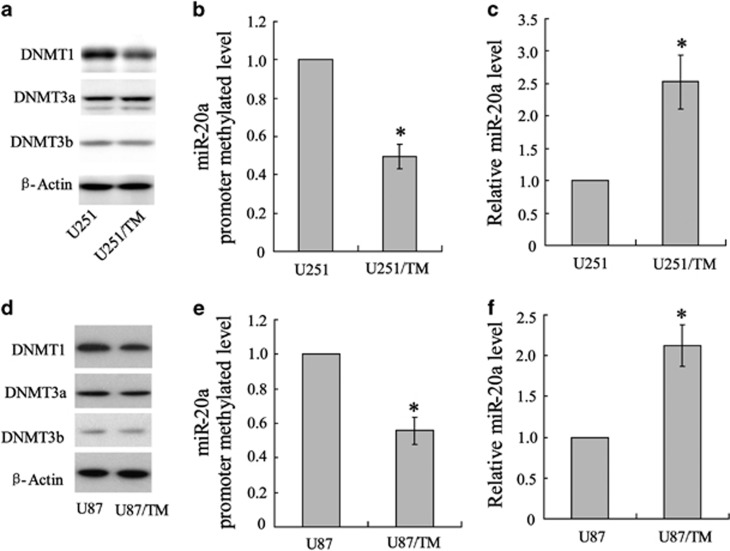
Characterization of miR-20a expression in glioma cells. The glioma cell lines U251 and U251/TM were used to determine (**a**) DNMT profiles using western blotting, (**b**) miR-20a promoter methylation using methylation-specific PCR (MS-PCR) and (**c**) miR-20a expression using quantitative RT-PCR. The glioma cell lines U87 and U87/TM were used to determine the expression of (**d**) DNMTs, (**e**) miR-20a promoter methylation and (**f**) miR-20a. Data were represented as the mean±s.d. *P<0.05 compared with the parental U87 or U251 cells.

The unsatisfactory outcome of chemotherapy with TMZ is defined primarily by intrinsic or acquired chemoresistance of GBM cells accompanied by a change in signal molecule expression. Transmethylase is a well-known epigenetic control element that regulates gene expression during chemoresistance.^5^ In this study, we chose two GBM cell lines and examined the effect of DNMT expression on TMZ sensitivity. As shown in Figures 1a and d, DNMT1 expression was downregulated, whereas levels of the DNMT3a and DNMT3b proteins were maintained in U251/TM cells compared with the parental U251 cells. To determine whether changes in miR-20a expression were involved in TMZ chemoresistance in glioma cells, the human miR-20a promoter was selected for methylation analysis. As shown in Figures 1b and e, proximal promoter methylation of miR-20a in U251/TM cells was decreased, resulting in an increase in miR-20a expression (Figures 1c and f).

### Methyltransferase inhibition reduced U251 TMZ sensitivity

**Figure 2:**
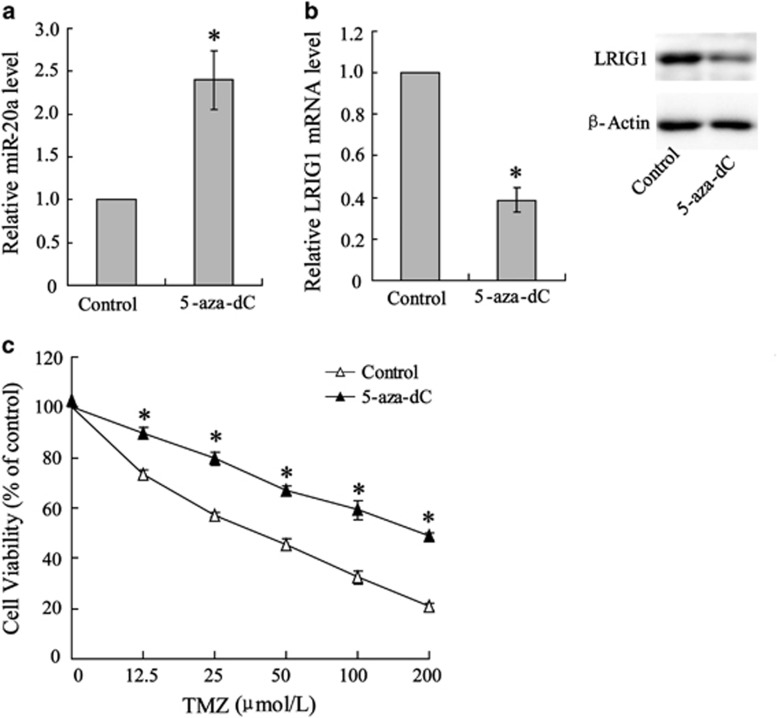
5-aza-dC treatment reduced the TMZ sensitivity of U251 cell. U251 cells were incubated with 5 μmol l^-1^ 5-aza-dC, a methyltransferase inhibitor prior to treatment with or without TMZ. (**a**) Quantification of miR-20a normalized to U6 RNA. (**b**) Quantification of LRIG1 mRNA normalized to NADPH and representation of LRIG1 protein expression. (**c**) Cell viability was determined using the MTT assay. Data were represented as the mean±s.d. *P<0.05 compared with the control.

As indicated above, miR-20a expression was promoted by a decrease in the methylation of its gene promoter; therefore, miR-20a might be involved in the development of chemoresistance in U251 cells. To examine this hypothesis, miR-20a expression was analyzed in U251 cells treated with 5-aza-dC (a methyltransferase inhibitor). As shown in Figure 2a, miR-20a expression showed a significant 2.5-fold increase in 5-aza-dC-treated U251 cells. In contrast, the expression of the LRIG1 gene that was negatively correlated with GBM chemoresistance^19^ was downregulated at both the mRNA and protein levels by 5-aza-dC treatment (Figure 2b). Subsequently, a MTT assay was performed to evaluate U251 cell viability; the result showed higher cell viability in 5-aza-dC-treated cells compared with the control (Figure 2c).

### Negative regulation of miR-20a expression by DNMT1 during the development of TMZ chemoresistance

**Figure 3:**
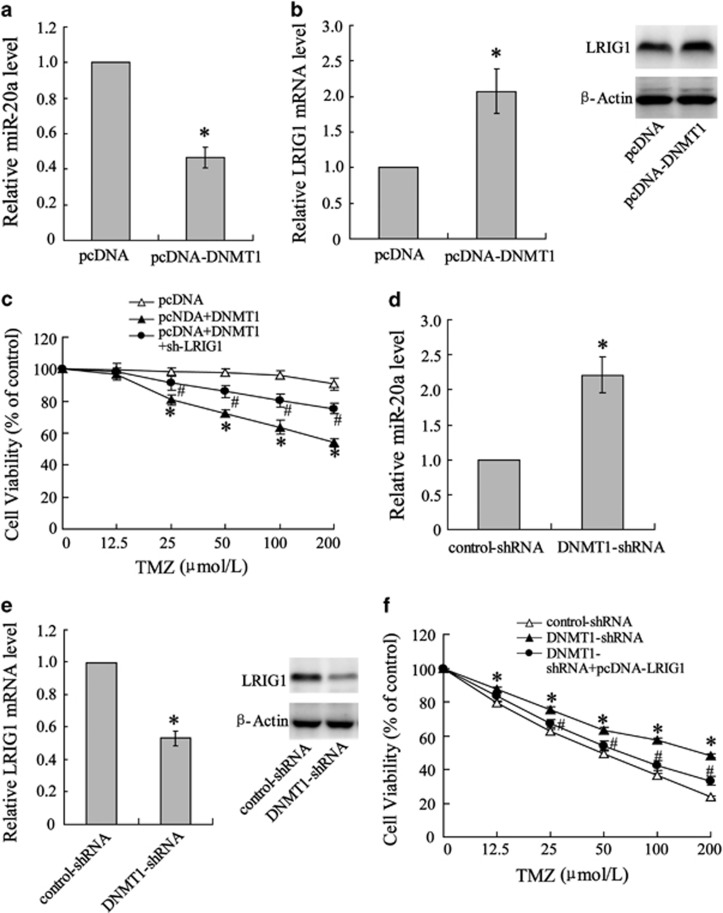
Negative correlation between DNMT1 and miR-20a expression in U251/TM cells. U251/TM cells were transfected with control pcDNA-DNMT1 or pcDNA and then lysed for detection of (**a**) miR-20a and (**b**) LRIG1 mRNA and protein expression. (**c**) Cells were co-treated with pcDNA-DNMT1 and sh-LRIG1, followed by a series of concentrations of TMZ. Then, cell viability was examined. U251/TM cells were transfected with DNMT1-shRNA or control-shRNA and then lysed for analysis of (**d**) miR-20a and (**e**) LRIG1 mRNA and protein expression levels. (**f**) Post-shRNA transfection, the cells were treated with a series of concentrations of TMZ and cell viability was examined. Data were represented as the mean±s.d. *P<0.05 compared with the control plasmid or negative control.

To investigate the regulation of the relationship between DNMT1 and miR-20a and whether their expression changes are correlated with TMZ chemosensitivity in U251 cells, overexpression of the DNMT1 protein was induced in U251/TM cells by transfection with pcDNA-DNMT1. The overexpression of DNMT1 suppressed miR-20a expression (Figure 3a), which was in agreement with the previous result in U251/TM cells. The transfection also increased LIRG1 expression at both the mRNA and protein levels (Figure 3b) and enhanced the TMZ sensitivity of the U251/TM cells in a manner that was abolished by sh-LRIG1 treatment (Figure 3c). In contrast, treatment of U251 cells with DNMT-shRNA to silence DNMT1 resulted in an increase in miR-20a expression (Figure 3d), decrease in LIRG1 expression (Figure 3e) and attenuation of TMZ sensitivity in U251 cells (Figure 3f). These data strongly confirmed the key regulatory role of DNMT1 in the development of glioma chemoresistance.

### miR-20a mediated DNMT1-stimulated LRIG1 expression

**Figure 4:**
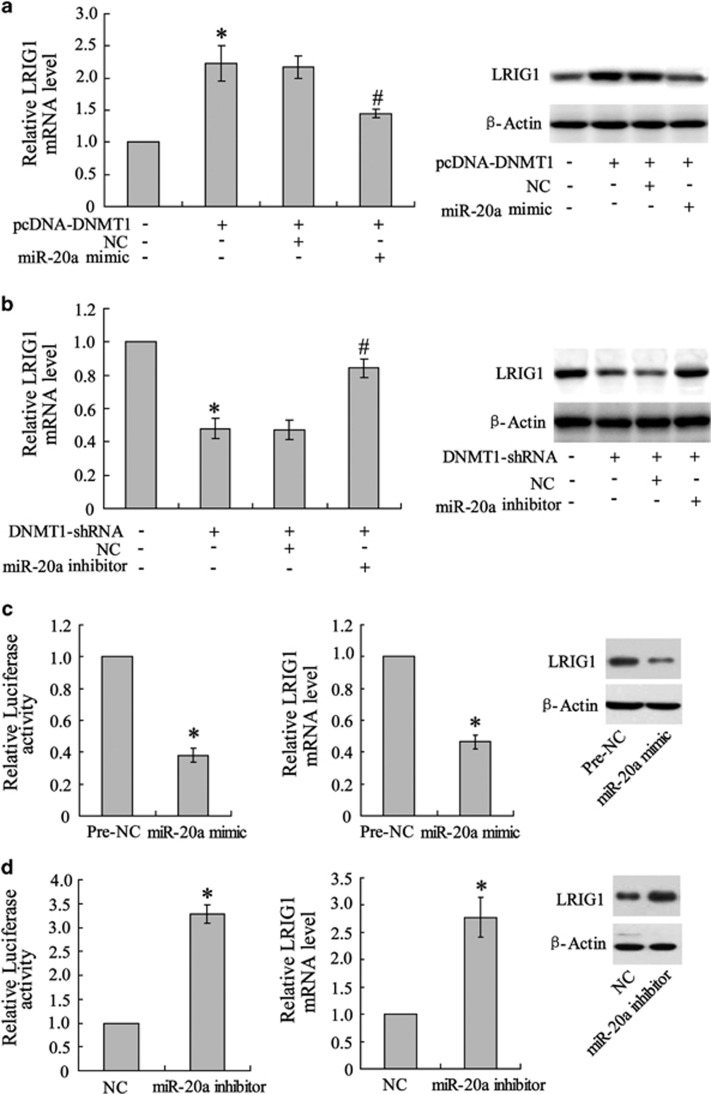
Abrogating effect of miR-20a on DNMT1-stimulated LRIG1 expression in U251/TM cells. (**a**) U251/TM cells were co-transfected with pcDNA-DNMT1 and the miR-20a mimic or its negative control (NC). Forty-eight hours post transfection, the cells were lysed and the LRIG1 mRNA and protein expression levels were determined. (**b**) U251/TM cells were co-transfected with DNMT1-shRNA and miR-20a inhibitor or its negative control (NC). Forty-eight hours post transfection, the cells were lysed and LRIG1 mRNA and protein expression levels were determined. (**c**) Cells were transfected with the miR-20a mimic or (**d**) miR-20a inhibitor, and 48 h post transfection the cells were transfected with the luciferase reporter gene plasmid that included the miR20-a binding site in the LRIG1 3′-UTR sequence. Luciferase activity and the mRNA and protein expression of LRIG1 were determined. Data were represented as the mean±s.d. *P<0.05 compared with the control plasmid, Pre-NC; ^#^*P*<0.05 compared with the negative control.

As indicated above, DNMT1 exerted a positive effect on LIRG1 expression in both U251/TM and U251 cells. To ascertain the role of miR-20a in DNMT1-stimulated LRIG1 expression, miR-20a was overexpressed in 251/TM cells treated with the miR-20a mimic prior to pcDNA-DNMT1 transfection. As shown in Figure 4a, miR-20a overexpression abrogated the DNMT1 overexpression-induced increase in LRIG1 expression at both the mRNA and protein levels. To confirm this phenomenon, miR-20a was silenced by its inhibitor in U251 cells prior to DNMT1-shRNA treatment. The results showed that the reduction in LRIG1 expression induced by DNMT1 silencing was reversed by miR-20a knockdown (Figure 4b). Next, we examined whether LRIG1 was the target gene for miR-20a. To this end, we transfected a LRIG1 3′-UTR luciferase reporter gene plasmid that included the miR-20a binding site into U251^miR-20a mimic^ and U251 ^miR-20a inhibitor^ cells. The results showed that LRIG1 3′-UTR activity and LRIG1 mRNA and protein expression were inhibited by the overexpression of miR-20a (Figure 4c) and enhanced by the knockdown of miR-20a (Figure 4d).

### miR-20a reversed TMZ-induced cell apoptosis via G1 phase retardation

**Figure 5:**
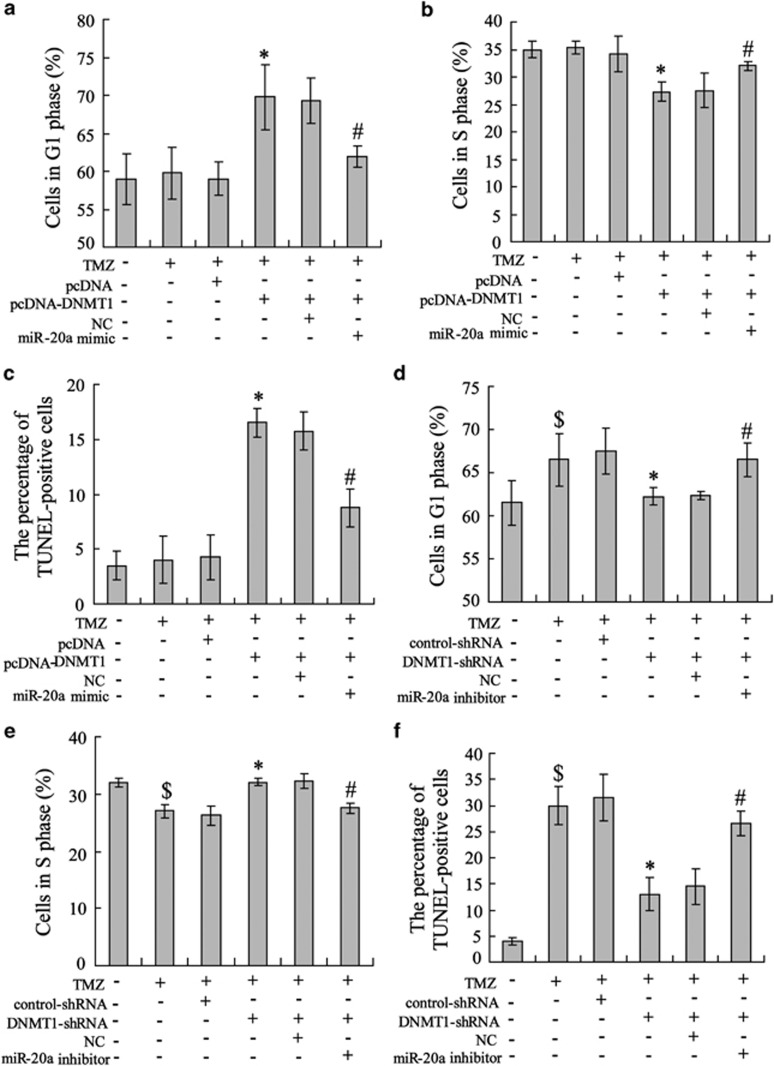
Abrogating effect of miR-20a on DNMT1-stimulated cell apoptosis in U251/TM cells. After transfection of U251/TM cells with pcDNA-DNMT1 and/or the miR-20a mimic, the cells were incubated with TMZ (5 μmol l^-1^) and lysed for analysis of (**a**) G1 phase and (**b**) S phase using the BrdU assay and (**c**) cell apoptosis using the TUNEL assay. U251/TM cells were transfected with DNMT1-shRNA and/or the miR-20a inhibitor prior to TMZ (5 μmol l^-1^) treatment. (**d**) The cells in the G1 phase, (**e**) S phase and (**f**) levels of cell apoptosis were determined as described above. Data were represented as the mean±s.d. ^$^*P*<0.05 compared with no TMZ treatment, *P<0.05 compared with the control plasmid+TMZ, ^#^*P*<0.05 compared with the negative control+TMZ.

The top functional networks between DNMT1 and miR-20a are related to TMZ resistance in U251 cells. Therefore, we attempted to determine their impact on TMZ-stimulated U251 cell proliferation. The cell cycle was analyzed after treatment of the cells with the corresponding regents. The results showed that DNMT1 overexpression increased the percentage of cells in G1 phase (Figure 5a) and decreased the percentage of cells in S phase (Figure 5b), thereby indicating a blockage of the U251/TM cell cycle at the G1-S phase transition that resulted in cell apoptosis (Figure 5c). This cell phenotype was reversed by co-transfection with pcDNA-DNMT1 and the miR-20a mimic (indicated on the right of Figures 5a–c). TMZ treatment in U251 cells increased the percentage of cells in G1 phase (left in Figure 5d), decreased the percentage of cells in S phase (left in Figure 5e) and promoted cell apoptosis. Subsequently, knockdown of DNMT1 by DNMT1-shRNA transfection led to a decrease in the percentage of cells in G1 phase (Figure 5d) and increase in the percentage of cells in S phase (Figure 5e). These data demonstrated an improvement in the reduction in cell survival following treatment with TMZ (Figure 5f). Moreover, this cell phenotype was reversed by co-transfection with DNMT1-shRNA and the miR-20a inhibitor (indicated on the right of Figure 5d–f).

### DNMT1 increased tumor sensitivity to TMZ in U251/TM xenografts

**Figure 6:**
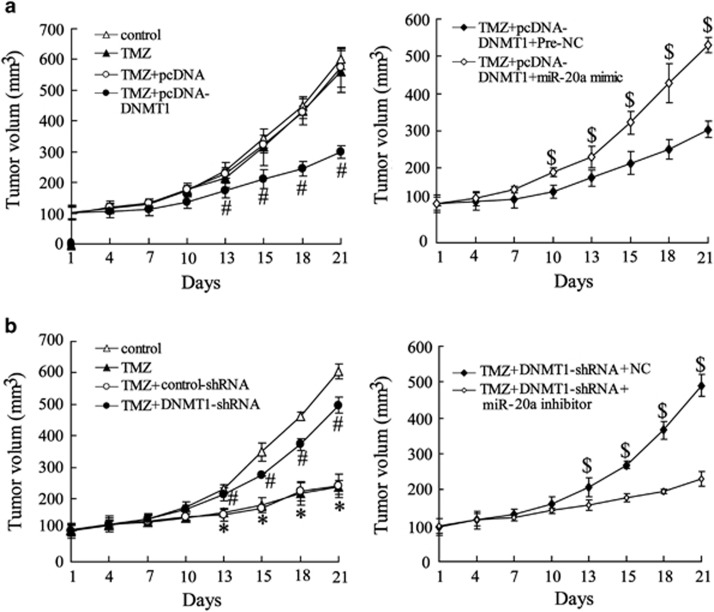
Effect of the DNMT1-miR-20a axis on tumor TMZ sensitivity in U251/TM xenografts. (**a**) Twenty-four hours after co-transfection with pcDNA-DNMT1 and/or the miR-20a mimic or pcDNA, U251/TM cells were transplanted into mice and tumor growth was measured from day 1 until day 21 post xenograft. During this time, the tumors were injected with TMZ (50 mg kg^-1^ per day) for 5 days when their volumes reached 0.5 cm. (**b**) Twenty-four hours after co-transfection with DNMT1-shRNA and/or the miR-20a inhibitor or it negative control, U251/TM cells were transplanted into mice. Tumor growth was measured from day 1 until day 21 post xenograft. During this time, the tumors were injected with TMZ (50 mg kg^-1^ per day) for 5 days when their volume reached 0.5 cm. Data were represented as the mean±s.d. *P<0.05 compared with the control, ^#^*P*<0.05 compared with plasmid+TMZ or negative control+TMZ; ^$^*P*<0.05 compared with pc3.1DNA-DNMT1 or sh-DNMT1.

To verify the key role of DNMT1 and miR-20a in U251 chemoresistance, U251/TM cells pretreated with pcDNA-DNMT1 and/or the miR-20a mimic were subcutaneously transplanted into the abdomens of mice. The resulting tumor was treated with TMZ (50 mg kg^-1^ day) for 5 days. The data showed that the tumors in the pcDNA-DNMT1 transfection group were smaller than those in the negative control+TMZ group on days 13–21, whereas the reduced tumor growth associated with DNMT1 overexpression was abrogated by treatment with the miR-20a mimic (Figure 6a). Tumors in the group transplanted with DNMT-shRNA-transfected U251/TM cells were larger than the tumors in the negative control+TMZ group on days 13–21, whereas the tumor growth induced by knockdown of DNMT1 was suppressed by miR-20a inhibitor treatment (Figure 6b).

## Discussion

TMZ treatment combined with radiotherapy and surgical resection improved both the overall survival and the progression-free survival in patients with newly diagnosed GBM.^1^ Because of its low toxicity, TMZ was the first chemotherapeutic agent reported to be suitable for long-term application over several years. Thus, improvement of TMZ chemoresistance would be greatly beneficial for GBM patients. TMZ-induced DNA methylation is often associated with the transcriptional repression of gene expression^20^ and decreased responsiveness to chemotherapy. We recently linked DNMT expression to TMZ resistance and glioblastoma cell survival, and further showed that DNMT1 downregulation led to changes in gene expression. These findings suggest that epigenetic control mechanisms are involved in TMZ resistance in GBM. In our current study, we demonstrated the specific contribution of an important epigenetic regulator (DNMT1) to the facilitation of miR-20a expression in chemoresistant glioblastoma cancer cells.

In mammals, global DNA methylation is catalyzed mainly by three DNMTs: DNMT1, DNMT3a and DNMT3b. Genetic and biochemical studies have suggested functional differences between these three enzymes despite the fact that they all contain highly conserved DNMT motifs.^21^ This hypothesis was confirmed by our observation that DNMT1 was downregulated under specific chemoresistance conditions, whereas the expression of DNMT3a and DNMT3b remained unchanged. DNMT1 (known as the maintenance DNMT) has a high preference for hemimethylated DNA substrates and is essential for maintaining methylation patterns and accurately replicating genomic DNA methylation patterns during cell division in mammalian cells.^22^ DNMT1 was demonstrated to exhibit a positive correlation with cancer cell chemoresistance in various tumors.^23^ Therefore, we evaluated its role in the development of glioma chemotherapy resistance. The results indicated that DNMT overexpression in U251/TM cells promoted TMZ-induced cell apoptosis by retarding cells in G1 phase. This finding was confirmed using an *in vivo* U251/TM cell xenograft that showed that DNMT1 overexpression inhibited tumor growth.

In addition to DNMT1 downregulation, glioma tumor promoter miR-20a^11^ expression was upregulated, thereby suggesting a possible association between transmethylase inhibition and miR-20a activity. To investigate this hypothesis, we treated U251 cells with 5-aza-dC (a transmethylase inhibitor); the results indicated a negative correlation between transmethylase inhibition and miR-20a expression. Previously, studies have focused on the mechanisms by which miRNAs are regulated in complex gene regulatory systems. For example, the genetic regulation of miRNAs is similar to mRNA regulation and involves the regulation of miRNAs by specific transcription factors or proteins that interact with the promoter.^12, 13^ Recent studies have shown that alterations in CpG island methylation in the regulatory regions of miRNAs affect miRNA expression and the regulation of downstream target mRNAs.^24, 25^ Using methylation-specific PCR, we observed a decrease in the miR-20a promoter methylation level that subsequently released miR-20a expression, leading to miR-20a aggregation in U251/TM cells.

miR-20a was capable of enhancing the invasiveness of CD133+ glioma stem cells (GSCs) isolated from both the U87 glioblastoma cell line and primary human glioma specimens.^26^ In addition, the miR-20a-mediated downregulation of NKG2D ligands contributed to glioma immune escape.^17^ Based on the observation that decreased DNMT1 expression was associated with increased miR-20a expression in physiological processes associated with glioblastoma chemoresistance, miR-20a was recognized as a potential key factor for DNMT1-controlled glioma chemoresistance. Indeed, DNMT1-induced changes associated with chemosensitivity were abrogated in U251 or U251/TM cells transfected with the miR-20a inhibitor or mimic. In addition, DNMT1 induced the expression of the LRIG1 protein. LRIG1 was shown to function as an inhibitor of receptor tyrosine kinases. Moreover, LRIG1 was demonstrated to be a tumor suppressor gene and may be related to glioma chemotherapy resistance, such as was observed in our present study,^16^ in a manner that could be reversed by miR-20a. Using a luciferase reporter gene assay, we observed that LRIG1 was a target gene of miR-20a. These data suggested that the miR-20a-LRIG1 axis was the downstream signaling target for the DNMT1 involved in the TMZ resistance of glioma cells.

From these studies, we propose that DNMT1 downregulation leading to TMZ resistance in glioma cells results in the demethylation of the miR-20a promoter, thereby causing the promotion of miR-20a expression and the development of chemoresistance. Thus, DNMT-mediated miR-20 expression may serve as a new potential therapeutic target for glioblastoma multiforme.
